# Assessment of soil water, carbon and nitrogen cycling in reseeded grassland on the North Wyke Farm Platform using a process-based model

**DOI:** 10.1016/j.scitotenv.2017.06.012

**Published:** 2017-12-15

**Authors:** Yuefen Li, Yi Liu, Paul Harris, Hadewij Sint, Phil J. Murray, Michael R.F. Lee, Lianhai Wu

**Affiliations:** a College of Earth Sciences, Jilin University, Changchun 130061, China; b Sustainable Soils and Grassland Systems Department, Rothamsted Research, North Wyke, Okehampton, Devon EX20 2SB, UK; c Key Laboratory of Aquatic Botany and Watershed Ecology, Wuhan Botanical Garden, Chinese Academy of Sciences, Wuhan 430074, China; d School of Veterinary Sciences, University of Bristol, Langford, Somerset BS40 5DU, UK

**Keywords:** SPACSYS, North Wyke Farm Platform, Reseeding, Soil water, Nutrient cycling

## Abstract

The North Wyke Farm Platform (NWFP) generates large volumes of temporally-indexed data that provides a valuable test-bed for agricultural mathematical models in temperate grasslands. In our study, we used the primary datasets generated from the NWFP (https://nwfp.rothamsted.ac.uk/) to validate the SPACSYS model in terms of the dynamics of water loss and forage dry matter yield estimated through cutting. The SPACSYS model is capable of simulating soil water, carbon (C) and nitrogen (N) balance in the soil-plant-atmosphere system. The validated model was then used to simulate the responses of soil water, C and N to reseeding grass cultivars with either high sugar (*Lolium perenne* L. cv. AberMagic) or deep rooting (*Festulolium* cv. Prior) traits. Simulation results demonstrated that the SPACSYS model could predict reliably soil water, C and N cycling in reseeded grassland. Compared to AberMagic, the Prior grass could fix more C in the second year following reseeding, whereas less C was lost through soil respiration in the first transition year. In comparison to the grass cultivar of the permanent pasture that existed before reseeding, both grasses reduced N losses through runoff and contributed to reducing water loss, especially Prior in relation to the latter. The SPACSYS model could predict these differences as supported by the rich dataset from the NWFP, providing a tool for future predictions on less characterized pasture.

## Highlights

• SPACSYS could simulate forage cutting dry matter, soil movement and nutrient cycling.
• Simulation showed that deep-rooting grass may reduce water loss from soil.
• Deep-rooting grass increased soil carbon storage from simulation results.
• Simulation demonstrated deep-rooting grass lowered soil denitrification loss.

## Graphical abstract

**Figure:**
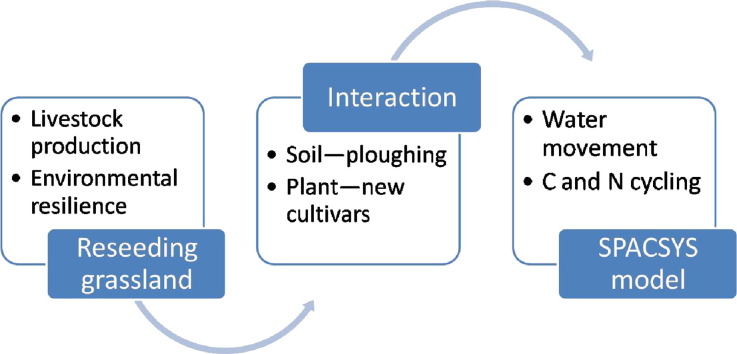

## 1 Introduction

The cycling of water, carbon (C) and nitrogen (N) have long been the three main components studied by ecosystem ecologists and global change scientists (Watanabe and Ortega, 2011). Grasslands are a crucial component of terrestrial ecosystems, covering 37% of the Earth's ice free land area, contributing to vital food production through grazing ruminants (O'Mara, 2012). In the UK, grasslands account for over 65% of the agricultural land (Humphreys et al., 2006, Vickery et al., 2001), providing a relatively cheap source of feed for ruminant livestock that in turn, offers highly nutritious meat and dairy products (Marshall et al., 2016). Grasslands have a high inherent soil organic matter (SOM) content that supplies nutrients to plants through decomposition and mineralisation, increases soil aggregation, limits soil erosion, and also increases cation exchange and water holding capacities (Conant et al., 2001), which could come from the interactions between the roots of different plant species and the soil in which they grow (Marshall et al., 2016). For example, grassland ecosystems may reduce soil erosion through a relatively stable and permanent plant cover and dense rooting systems that maintain soil cover (Conant et al., 2001) and sequester more C (Ostle et al., 2009). Alongside this, grassland tends to have a higher turnover of root and leaf material than in other ecosystems, thus favouring a relatively high level of soil fertility (Rumpel et al., 2015). However, yields and quality of permanent grasslands tend to decrease over time due to ageing (Velthof et al., 2009), where this can be rapid following extreme weather conditions and/or poor grassland management decisions (Semmartin et al., 2010) that can alter the botanical composition of the sward. In the UK, grassland management has changed substantially during the second half of the 20th century, one of which is that structurally diverse and species-rich swards have been largely replaced by relatively dense, fast-growing and uniform swards (Vickery et al., 2001). However, there might be a trend that intensive grasslands are being reversed back to multi species swards with a proportion of legumes.

Intensive grassland management may lead to uncoupling of nutrient cycles (Dungait et al., 2012). Murray et al. (2012) reported interactions between the plant and the soil were crucial in regulating soil processes for the perennial permanent pasture. In order to increase sward productivity, which also have the potential to increase soil C and N contents, a variety of management techniques are used including: fertilization (inorganic and organic), aeration, grazing management (e.g. strip grazing), earthworms, and sowing of favourable forage grasses and legumes (Bilotta et al., 2007, Knight et al., 1992, Peukert et al., 2016). Planned and regular reseeding as an important management system not only maintains sward productivity but improves a range of ecosystem services, such as genetic conservation (Firbank et al., 2013), prevention of soil erosion due to reseeding ‘ideal’ species possessing deep and extensive root systems with fast-growing but strong roots (Stokes et al., 2009), and prospectively enhancing long-term soil C storage. However, such management may cause severe soil organic C and N loss in the reseeding phase, for example, Necpálová et al. (2013) reported that ploughing of grassland led to physical disruption of soil structure, increased aeration, and consequently accelerated soil mineralization processes. Therefore, a systems approach to assess quantitatively the impact of reseeding new cultivars on nutrient cycling and water redistribution is still needed.

The fluxes of energy, water, C and N through soil-plant-atmosphere systems are closely coupled and complex (Grant, 1995, Thornley et al., 1995). Modelling is an efficient tool to investigate the impact of external disturbance (agronomic practices and unprecedented weather events) on plant growth, nutrient cycling and water movement in the systems. Shepherd et al. (2011) reviewed the applicability of thirty models given a set of essential criteria related to scale, biophysical processes, and land management. They concluded that although no single model incorporates all criteria, SPACSYS, together with DAYCENT and PASIM, could all consider a water balance and water movement through soil to simulate nutrient cycling processes, specifically C and N dynamics at the field scale with the change of agricultural practices relating to crop and livestock management. Previous studies suggested that the SPACSYS model could reliably simulate N_2_O emissions from grasslands (Abalos et al., 2016, Wu et al., 2015) and other systems (Perego et al., 2016, Zhang et al., 2016), water fluxes and grass growth (Wu et al., 2016), and crop yield and soil C and N stocks under climate change scenarios with fertiliser management (Zhang et al., 2016). For these reasons and more, the SPACSYS model is ideally suited to the simulation objectives of this study.

**Fig. 1:**
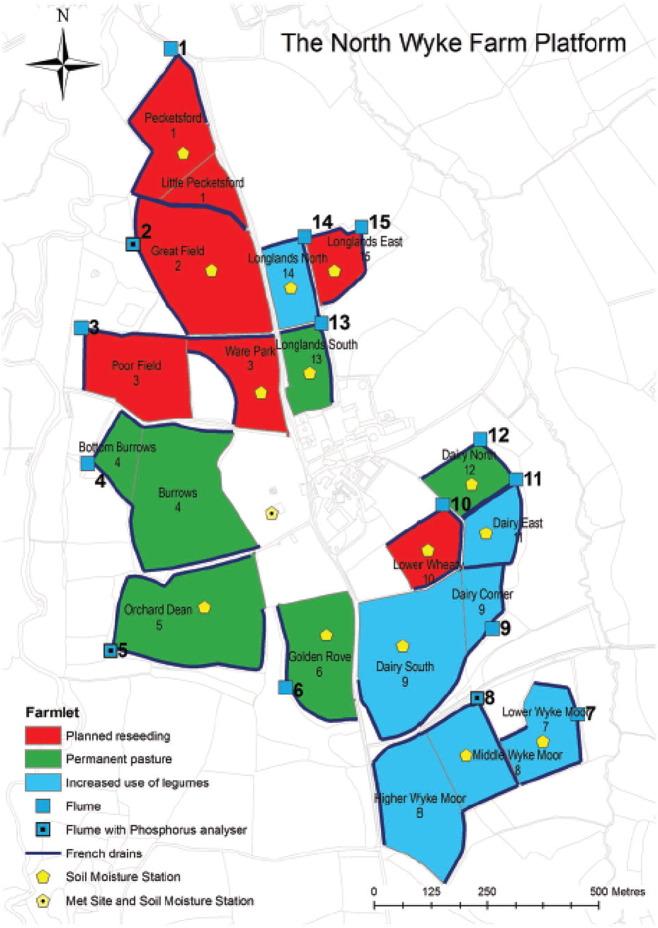
Distribution of grassland management fields and sub-catchments of the North Wyke Farm Platform. This study's reseeding fields/sub-catchments constitute the ‘red’ farmlet.

The North Wyke Farm Platform (NWFP) farm-scale experimental system was established in 2010 as a UK national capability and its remit is to research agricultural productivity and ecosystem responses to different management practices for beef and sheep production in lowland temperate grasslands (Orr et al., 2016). The NWFP provides three farming systems (farmlets): (i) permanent pasture (‘green’ farmlet), (ii) grass and white clover lays (‘blue’ farmlet) and (iii) an improved grass sward through planned regular reseeding (‘red’ farmlet). The ‘red’ farmlet on the platform is managed to take advantage of grass germplasm improvement through regular renewal (about every 4 years), providing opportunities for introducing innovative cultivars with desirable traits. Each farmlet consists of five hydrological isolated sub-catchments comprising approximately 21 ha. The sub-catchments were allocated to each farmlet primarily based on: 1) historical farm practices; 2) expert knowledge of the physical properties of the North Wyke site; 3) spatial connectivity between the five sub-catchments of each farmlet and 4) farm/research operational requirements. Six of the 15 sub-catchments have field divisions, providing 21 fields in total across the NWFP (Fig. 1).

In this study, the data collected from five sub-catchments (and seven fields) of the reseeding ‘red’ or ‘innovation’ farmlet of the NWFP were used to validate the SPACSYS model and to further investigate the effects of reseeding grass varieties with different traits on water movement and nutrient cycling. In doing so, this study contributes to the understanding of a grassland reseeding process with attention given to N losses from the plant-soil system over a five-year model simulation period. In turn, this helps to identify a suitable variety to be reseeded in order reduce nitrate leaching while sequestering the relevant amount of C to the soil.

## 2 Materials and methods

### 2.1 Research area and experimental design

The NWFP is located on the Rothamsted Research, North Wyke Farm in southwest England (50°46′10″N, 3°54′05″W). The mean annual rainfall between 1985 and 2015 was 1033 mm, with an annual average temperature that ranged from 6.8 to 13.4 °C. The soil is predominantly of two similar series, Hallsworth and Halstow, that comprise of a slightly stony clay loam topsoil (approximately 36% clay) overlying a mottled stoney clay (approximately 60% clay), derived from carboniferous culm measures (Orr et al., 2016). The soils at North Wyke are representative of many areas in western England, and are shallow and cover highly impermeable clay-layers, and are thus prone to flooding (Harrod and Hogan, 2008).

For the study period in this paper, two cultivars were sown: diploid perennial ryegrass, AberMagic (*Lolium perenne* L.), bred to express high levels of fructan (sugar) to improve energy and protein provision to grazing ruminants (Lee et al., 2001) and a hybrid cross between the *Festuca* and *Lolium* species (Staerfl et al., 2012), *Festulolium* cultivar Prior, with deep rooting traits to improve drought resistance and aid water abatement. The permanent pasture (all using the same cultivar) was replaced with the new cultivars within the ‘red’ study farmlet as planned in Table 1. Observe that the time between ploughing and reseeding was quite variable across the seven fields. This was an unavoidable artefact of the farm management operations and was assumed not to have an adverse effect on this study's results with respect to C and N cycling. Each NWFP farmlet ran a beef (30 weaned cattle; Hereford-Friesian × continental) and sheep (75 ewes and their lambs; Suffolk-Mule × Charollais) operation where animals were continuously set-stocked based on sward surface height with surplus forage cut for silage preservation as winter feed.

**Table 1:** Dates of ploughing and reseeding for individual fields.

Field name	Sub-catchment number	Area (ha)	Ploughing date	Reseeding date	Reseeding cultivar
Great Field	2	6.65	6th July 2013	30th July 2013	AberMagic
Longlands East	15	1.54	10th July 2013	7th August 2013	Prior
Poor Field	3	3.92	25th July 2014	21st August 2014	AberMagic
Ware Park	3	2.71	25th July 2014	21st August 2014	AberMagic
Pecketsford	1	3.52	29th July 2015	11st August 2015	AberMagic
Little Pecketsford	1	1.30	29th July 2015	7th August 2015	AberMagic
Lower Wheaty	10	1.82	3rd August 2015	11st August 2015	AberMagic

### 2.2 SPACSYS model

The SPACSYS model is a process-based, field scale, weather-driven and daily-time-step dynamic model to simulate plant growth and development, soil C and N cycling, water dynamics and heat transformation (Wu et al., 2007, Wu et al., 2015). N cycling coupled with C cycling in the model covers the transformation processes for organic matter and inorganic N plus a biological-based component for the denitrification process that can estimate N gaseous emissions. The main processes concerning plant growth are assimilation, respiration, water and N uptake, partition of photosynthate and N, N-fixation for legume plants and root growth. The Richards equation for water potential is used to simulate water and heat fluxes. As the model has been described in detail elsewhere, only the relevant model input data and simulation output variables which are used for comparing with observed data are presented here.

### 2.3 Model input and parameterisation

Site-specific input data for the simulations include daily weather data, soil properties, field and grass management (e.g. fertiliser application date and composition, reseeding, grazing and cutting dates), and initialization of the state variables (standing biomass and root distribution, soil water and temperature distribution). Daily weather data recorded at the North Wyke site were used. Soil physical and chemical properties of the fields were based on a baseline field survey conducted in summer 2012 (Orr et al., 2016, Table A1). Agronomic management quantified for the simulations were interpreted from the farm records for the NWFP (https://nwfp.rothamsted.ac.uk/). To mimic grazing systems, daily grass intake and excretion of sheep and cattle in the field were pre-processed and treated as agronomic management. The parameters used to estimate inputs to various soil C and N pools and grass intake in the seven fields of the farmlet followed Wu et al. (2016). The SPACSYS model has been parameterized previously for the processes of soil water and heat transformation, grass growth and C and N cycling on the NWFP (Wu et al., 2016). These parameters were again used directly in the simulations. Differences in the parameters between the two cultivars are listed in the Appendix (Table A2).

### 2.4 Model simulations

The SPACSYS model was run to predict grass growth, C and N cycling and water redistribution over the simulation period (2011–2015) for each field of the ‘red’ reseeding farmlet. Simulations were run for two years of permanent pasture prior to the start of the NWFP experiment to reduce side-effects of initial contents of soil C, N and water. Removal forage biomass for silage from the fields and water fluxes collected from the corresponding sub-catchment water flume over the period were used to validate the model, using data from https://nwfp.rothamsted.ac.uk/. Cutting biomass were measured 1–4 times for the seven reseeding fields over the period using a Haldrup forage harvester prior to silage cut.

### 2.5 Statistical analyses

The following diagnostics were used to evaluate model performance by comparing the SPACSYS simulated data with the measured data: (i) the correlation coefficient (r), (ii) the root mean square error (RMSE), (iii) the modelling efficiency (EF), (iv) the relative error (RE), (v) the mean deviation (MD), (vi) the maximum error (ME), and (vii) the coefficient of determination (CD). These diagnostics are a subset of that proposed by Smith et al. (1997) for evaluating process-based models.

## 3 Results

### 3.1 Model validation

**Fig. 2:**
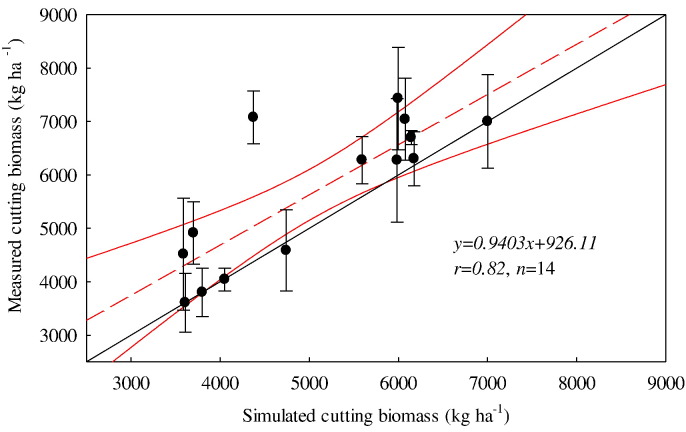
Comparison of simulated and measured cutting forage biomass from 2011 to 2015, excluding data from the first cut after reseeding. Red dashed line shows the fitted relationship; red solid lines are 95% confidence intervals; black solid line is 1:1 line; error bars are standard deviations for measured data.

Simulated cutting biomass from all fields selected over the simulation period was compared with measured data (Fig. 2). Cutting forage dry matter from the first cutting following reseeding was excluded as it has been demonstrated that the SPACSYS model over-predicts the first cutting biomass after reseeding (Wu et al., 2016). The resultant statistical analysis suggested that the simulation data correlates to the measured data reasonably well (Table 2).

**Table 2:** Statistical analysis of simulated and measured cutting forage biomass (data from the first cut after reseeding were excluded).

r	RMSE_95%_^a^	EF	CD	RE_95%_^b^	MD	ME	Number of samples
0.82^c^	17.29	0.45	1.02	9.90	623.80	2703.27	14

a RMSE at the 95% confidence level.

b RE at the 95% confidence level.

c Significant association at the 5% level.

**Fig. 3:**
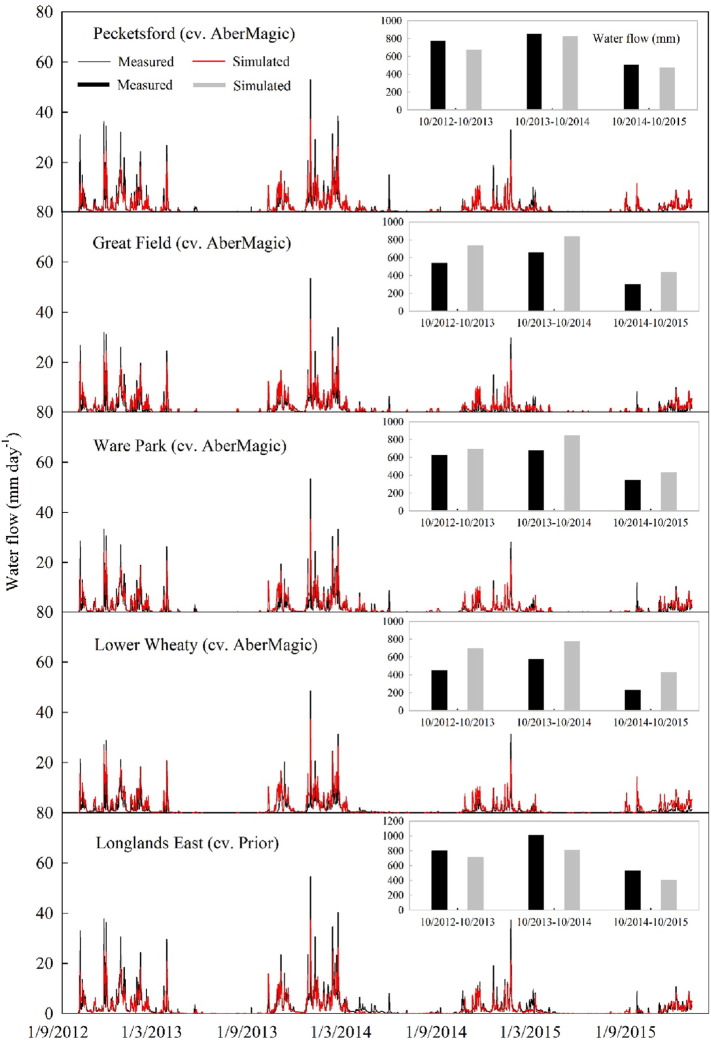
Comparison of simulated and measured water flow from October 2012 to December 2015. The inset histograms illustrate the annual comparison of simulated and measured water flow.

The general trend and patterns of the simulated fluxes tends to match that of the measured data (Fig. 3), with correlation coefficients of r > 0.86, and values for EF and CD close to 1, in all five cases (Table 3). Furthermore, the simulated fluxes could identify the measured peak flows reasonably well, which is vital to the capturing of unusual or extreme events. The fluxes were generally over-predicted for the sub-catchments/fields where AberMagic was reseeded, except for sub-catchment 1 (Pecketsford with Little Pecketsford), where simulated fluxes were lower on average. Further, the model under-predicts water fluxes in the Longlands East sub-catchment where Prior was reseeded.

**Table 3:** Statistical analysis of simulated and measured water flow for the five sub-catchments (Total number of samples for each sub-catchment is 999).

Sub-catchment	No. 1	No. 2	No. 3	No. 10	No. 15
r	0.90^a^	0.90^a^	0.90^a^	0.86^a^	0.92^a^
RMSE_95%_	98.26%	119.82%	113.25%	154.56%	88.04%
EF	0.8	0.8	0.8	0.7	0.82
CD	1.58	1.14	1.23	0.84	1.65
RE_95%_	− 353.39	− 339.17	− 331.4	− 533.74	− 187.06
MD	0.19	− 0.49	− 0.31	− 0.59	0.43
ME	19.72	9.46	13.8	10.8	15.9

a Denotes significant association at the 5% level.

### 3.2 The effects of reseeding on soil water balance

**Fig. 4:**
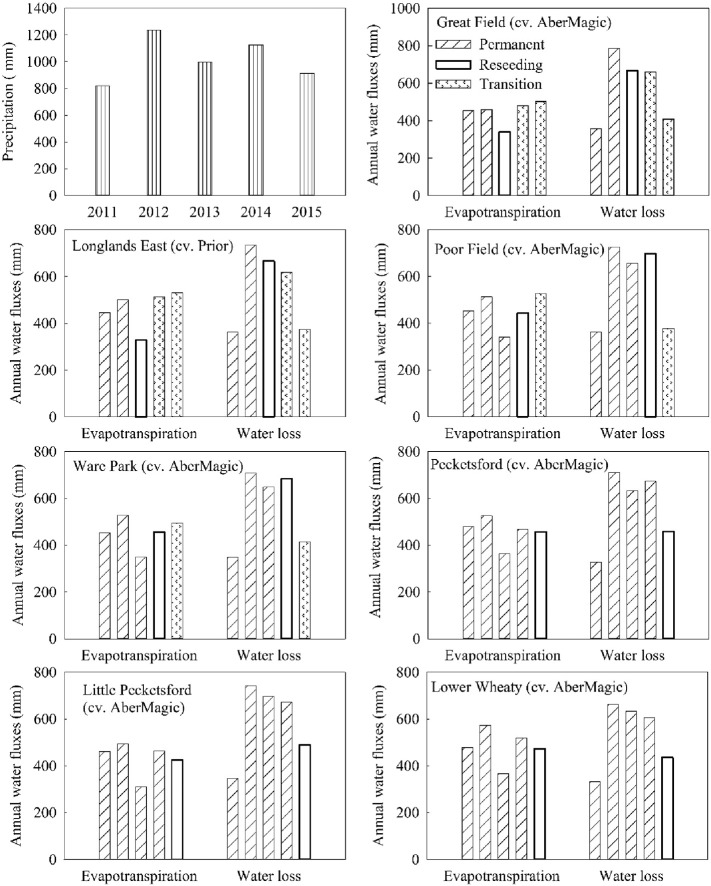
Simulated average annual fluxes of water from the reseeding fields. Five columns in each group represent each year from 2011 (left) to 2015 (right). The precipitation of all reseeding fields was the same.

Simulated water fluxes increased in the reseeding years compared to that from the permanent fields in same year. However, the water loss declined in the transition years following reseeding (Fig. 4). These simulations demonstrate that new established grasses will tend to stabilize after two years and the new grass cultivars, especially Prior, will contribute to reducing water loss. Annual evapotranspiration was generally greater for the transition period than for permanent grassland and Prior increased annual evapotranspiration compared with AberMagic.

### 3.3 The effects of reseeding on C and N cycling

The impact of reseeding on soil C and N stocks in the top 1.5 m of soil was different from field to field (Table 4). For the fields where AberMagic was reseeded, there was a trend for lower soil C and N storage after reseeding in Great Field, but was more idiosyncratic for Poor Field and Ware Park (Table 4). Because the simulation period after reseeding was less than one year in Pecketsford, Little Pecketsford and Lower Wheaty, we were unable to compare the change of soil C and N stocks before and after reseeding. However, the average annual change rate in soil C showed an increase at 0.1–0.32%, while for soil N it deceased at ca. 0.04–0.19% (Table 4). Soil C storages in Longlands East where Prior was reseeded showed a small but persistent rise (from 95.01 to 96.06 t C ha^− 1^), whereas soil N storage had a reduction at the end of the first year after reseeding which rebounded in the second year.

**Table 4:** Simulated soil C (t C ha^− 1^) and N storage (t N ha^− 1^) in the top 1.5 m of soil and their change rates (%) compared with that before reseeding.

		Great Field (cv. AberMagic)		Longlands East (cv. Prior)	
		Storage	Change rate	Storage	Change rate
Soil C	Before reseeding	99.41	–	95.01	–
	1 year after reseeding	98.01	− 1.42	96.04	1.08
	2 years after reseeding	97.38	− 2.04	96.06	1.11
Soil N	Before reseeding	12.04	–	11.72	–
	1 year after reseeding	11.95	− 0.71	11.65	− 0.57
	2 years after reseeding	12.01	− 0.25	11.76	0.34
		Poor Field (cv. AberMagic)		Ware Park (cv. AberMagic)	
Soil C	Before reseeding	128.96	–	94.09	–
	1 year after reseeding	128.35	− 0.47	94.39	0.31
Soil N	Before reseeding	15.71	–	11.76	–
	1 year after reseeding	15.82	0.66	11.79	0.28
		Pecketsford (cv. AberMagic)		Little Pecketsford (cv. AberMagic)	
Soil C	Before reseeding	126.71	0.32	134.36	0.10
Soil N	Before reseeding	16.13	− 0.04	16.98	− 0.19
		Lower Wheaty (cv. AberMagic)			
Soil C	Before reseeding	146.76	0.12		
Soil N	Before reseeding	17.90	− 0.11		

**Fig. 5:**
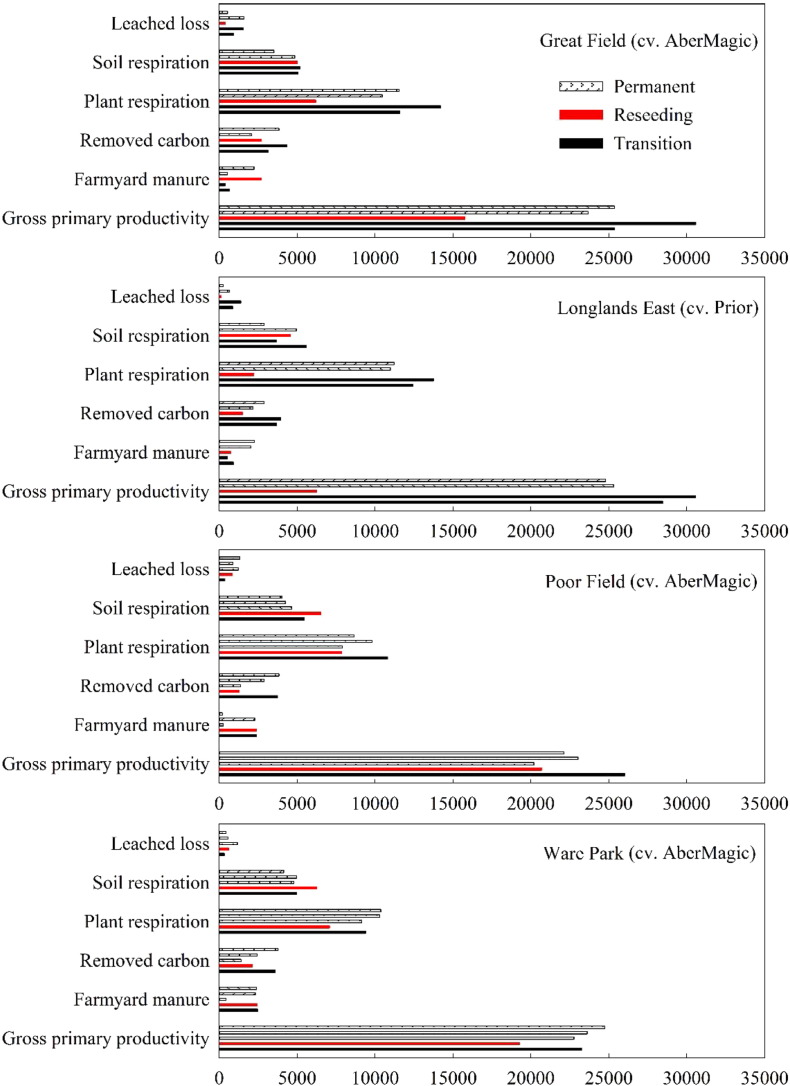

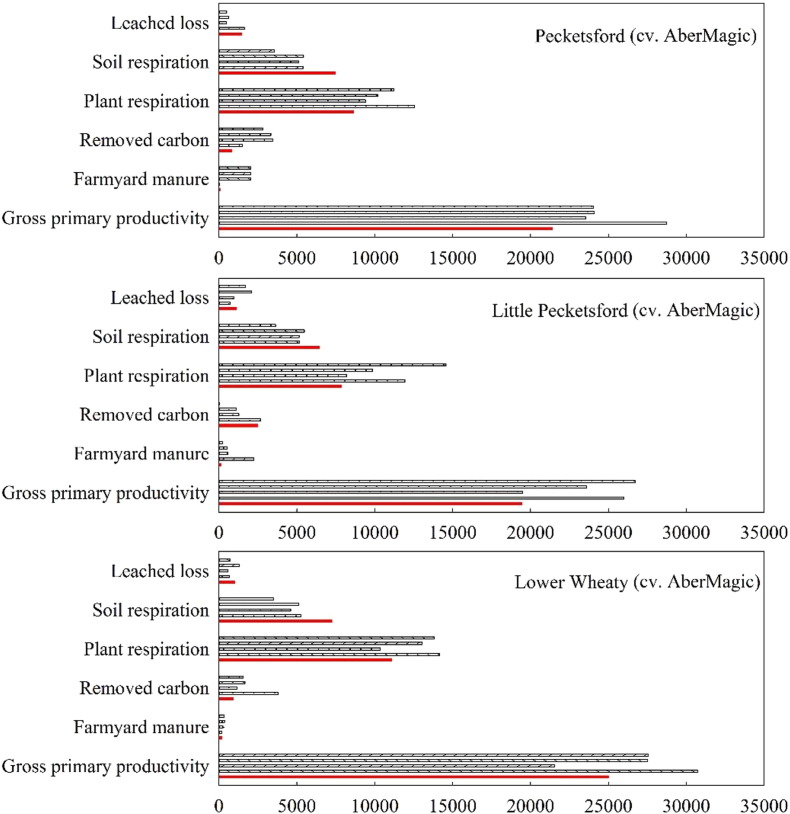
Simulated average annual fluxes of C (kg C ha^− 1^) from the reseeding fields. Five columns for each group on the y-axis represent each year from 2011 (top) to 2015 (bottom).

Reseeding led to a decrease in fixed C by grass (termed gross primary productivity in Fig. 5) during the reseeding year. However, after one-year establishment, more C was fixed (Fig. 5). Prior had significantly more fixed C than AberMagic in the second transition year, and resulted in significantly lower soil respiration when compared to AberMagic grass only in the first transition year.

**Fig. 6:**
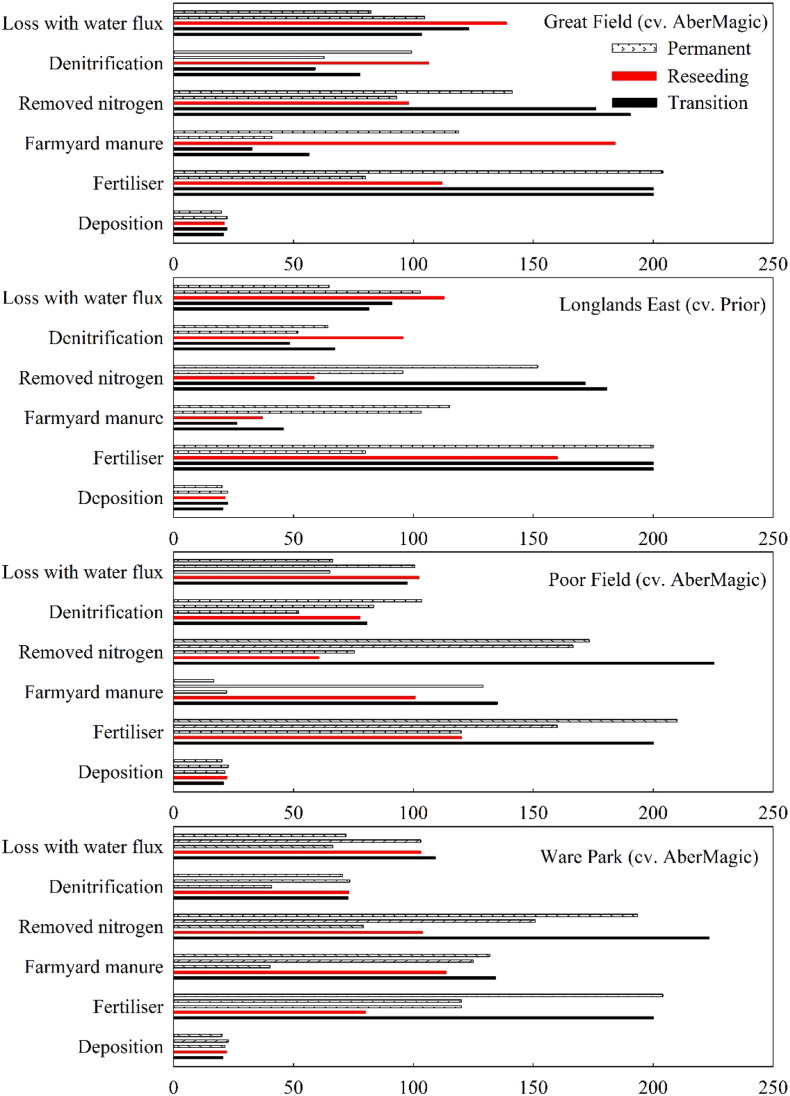

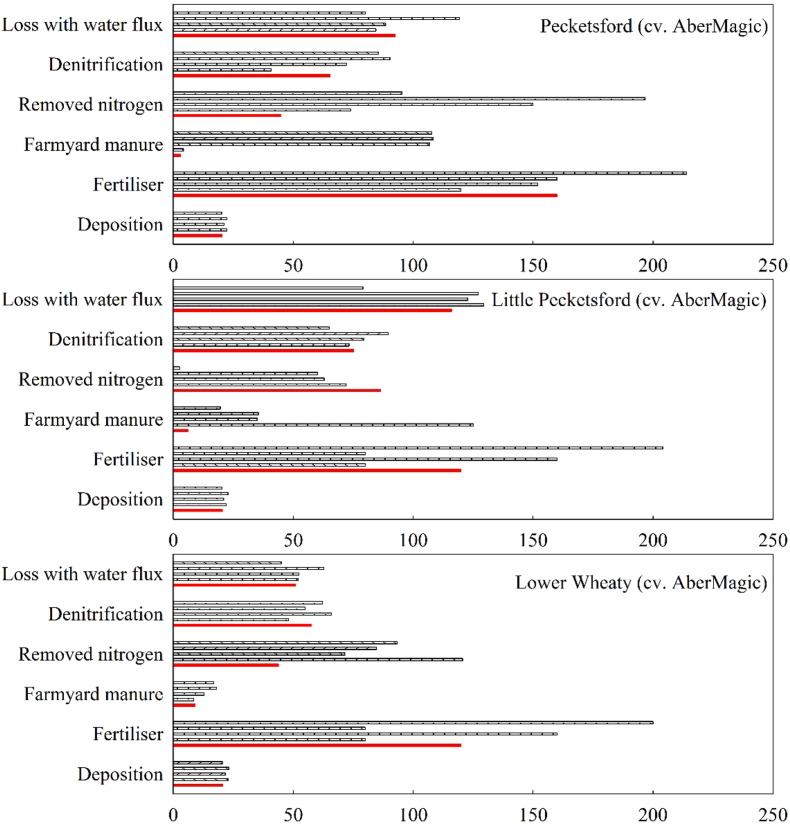
Simulated average annual fluxes of N (kg N ha^− 1^) from the reseeding fields. Five columns for each group on the y-axis represent each year from 2011 (top) to 2015 (bottom).

Fig. 6 shows the average annual fluxes of N from the seven reseeding fields before and after ploughing and reseeding. Among all the N output components, removed N and leached loss were the important components for all the fields, accounting for over 60% of the total N loss. The second N loss was denitrification, accounting for about 15–35%. The lowest N loss was lost through runoff. AberMagic or Prior reseeding resulted in higher N losses in the transition year compared to permanent grassland, whereas lower runoff loss occurred in the second transition year. Prior caused lower denitrification loss during transition, as compared to AberMagic.

## 4 Discussion

The validation results demonstrated the SPACSYS model parameters to be representative of processes that occur in a reseeded grassland, and thus SPACSYS could effectively assess soil water, C and N cycling in seven reseeded fields of the NWFP. The model could also adequately simulate cutting forage dry matter (Fig. 2 and Table 2). Nevertheless, and as would be expected, discrepancies still occurred between measurements and simulations. The reasons for this might be related to the partitioning coefficients of daily photosyntheate (Wu et al., 2016). The peak flows of water flux simulated by SPACSYS was generally smaller than that measured (Fig. 3). This was consistent with previous research (Ahn et al., 2014) in which it has been demonstrated that a model with long time steps (e.g. daily time step) tends to smooth simulated peak flows. Since all reseeding fields of this study had been established for just 1–2 years by the end date of the simulations, the systems may not yet have fully stabilized following the soil disturbance. Therefore, further validation of the effect of reseeding on soil water, C and N cycling using the SPACSYS model with a longer simulation period may further improve its predictive capability.

The simulations clearly showed that reseeding grassland affected soil water movement, C and N cycling. Reseeding brought about increases of water and N losses and soil respiration, together with decreases of fixed C, soil C and N storage in the reseeding year. These results concur with various experiments conducted under similar weather condition and soil type, to that studied here (Carolan and Fornara, 2016, Scholefield et al., 1993, Shepherd et al., 2001). Because of ploughing and associated reseeding, such soil disturbance will inadvertently cause water loss and losses of C and N into the environment. However, different cultivars showed different effects on water movement during the transition years (Table 5). For example, Prior reduced water loss (376.15 mm) in the second transition year following reseeding. From the reseeding year to the second transition year, the runoff coefficients (the ratio of water loss to precipitation) were 0.67, 0.55 and 0.41, respectively. Simulations showed that reseeding with Prior had achieved the better soil water retention effect. This may be attributed to its deep rooting architecture. Deep-rooting plants generally have larger water storage capacity compared to shallow rooting plants, which can combat flooding and reduce soil erosion (Loades et al., 2010, Macleod et al., 2013).

**Table 5:** The runoff coefficients of the seven reseeding fields from 2011–2015.

Field	2011	2012	2013	2014	2015
Great Field (cv. AberMagic)	0.43	0.64	0.67 (R)	0.59 (T1)	0.45 (T2)
Longlands East (cv. Prior)	0.44	0.60	0.67 (R)	0.55 (T1)	0.41 (T2)
Poor Field (cv. AberMagic)	0.44	0.59	0.66	0.62 (R)	0.41 (T1)
Ware Park (cv. AberMagic)	0.42	0.57	0.65	0.61 (R)	0.45 (T1)
Pecketsford (cv. AberMagic)	0.40	0.58	0.63	0.60	0.50 (R)
Little Pecketsford (cv. AberMagic)	0.42	0.60	0.70	0.60	0.54 (R)
Lower Wheaty (cv. AberMagic)	0.40	0.54	0.63	0.54	0.48 (R)

Symbols in the parentheses indicate reseeding year (R), the first transition year (T1) and the second transition year (T2), respectively. Table entries without any parentheses are for a permanent pasture year.

The simulations showed that the modelled soil C storage with reseeded Prior was gradually built up from the reseeding year (Table 4) and increased 0.525 t C ha^− 1^ in two years following reseeding. Furthermore, Prior could increase C fixation in the second transition year in the absence of significant differences in the quantity and quality of soil C inputs between AberMagic and Prior. The main reason for increasing soil C stocks is that Prior has greater root biomass and is proportionately deeper rooting. Similar results have been reported, where reseeding with improved species could increase C in a plant-soil system (Humphreys et al., 2014). However, the C loss from Longlands East also increased through soil respiration in the second transition year (1.53 g C m^− 2^ d^− 1^) compared with other years (0.79–1.35 g C m^− 2^ d^− 1^). This was likely a result of root exudation as a C substrate and the wide range of metabolites that they release to the soil (Pierret et al., 2016).

The simulations suggested that reseeding results in higher N loss through leaching, surface loss through runoff, and denitrification in the transition year, compared to the permanent grassland (Fig. 6). It has been reported that, in the case of immediately reseeding new cultivars, there is a period with no or only a small grass N uptake (Necpálová et al., 2013). The mineralized and applied N may exceed the amount grass requires, and lead to considerable losses from soils, particularly with high moisture conditions. Reseeding deep rooting grass (Longlands East, 78.25 kg N ha^− 1^ yr^− 1^) showed smaller amounts of N leached losses compared with the AberMagic (Great Field, 101.66 kg N ha^− 1^ yr^− 1^) in the second transition year, but it was opposite for N loss through surface runoff. The reason could be that deeper and bushy root ecosystems improve simultaneously both the soil structure and the retention of water and nutrients (Kell, 2011). High sugar grass has high yield so there are significant environmental benefits to decrease N runoff loss. Furthermore, high sugar grasses have been shown to increase N capture in the ruminant through enhanced microbial protein synthesis in the rumen, reducing N loss from livestock to pasture in the form of urine and faeces (Lee et al., 2002, Merry et al., 2006).

## 5 Conclusions

The SPACSYS model provided reasonable simulations on cutting forage biomass and the dynamics of water fluxes when compared to the primary measured data collected from the reseeded fields of the ‘innovation’ farmlet of the North Wyke Farm Platform experiment. The simulation results suggest that reseeding affects soil water redistribution in the soil profiles, nutrient cycling, and plant productivity. In addition, deep-rooting grasses appear conducive to reducing water loss and to sequestering more C into the soil. Moreover, deep-rooting grass might reduce denitrification during the transition years compared to high sugar grass.

## Appendix A Supplementary data

Supplementary tables Image 2

## References

[bb0005] Abalos D., Cardenas L.M., Wu L. (2016). Climate change and N_2_O emissions from South West England grasslands: a modelling approach. Atmos. Environ..

[bb0010] Ahn J., Cho W., Kim T., Shin H., Heo J.-H. (2014). Flood frequency analysis for the annual peak flows simulated by an event-based rainfall-runoff model in an urban drainage basin. Water.

[bb0015] Bilotta G.S., Brazier R.E., Haygarth P.M. (2007). The impacts of grazing animals on the quality of soils, vegetation, and surface waters in intensively managed grasslands. Adv. Agron..

[bb0020] Carolan R., Fornara D.A. (2016). Soil carbon cycling and storage along a chronosequence of re-seeded grasslands: do soil carbon stocks increase with grassland age?. Agric. Ecosyst. Environ..

[bb0025] Conant R.T., Paustian K., Elliott E.T. (2001). Grassland management and conversion into grassland: effects on soil carbon. Ecol. Appl..

[bb0030] Dungait J.A.J., Cardenas L.M., Blackwell M.S.A., Wu L., Withers P.J.A., Chadwick D.R., Bol R., Murray P.J., Macdonald A.J., Whitmore A.P., Goulding K.W.T. (2012). Advances in the understanding of nutrient dynamics and management in UK agriculture. Sci. Total Environ..

[bb0035] Firbank L., Bradbury R.B., McCracken D.I., Stoate C. (2013). Delivering multiple ecosystem services from enclosed farmland in the UK. Agric. Ecosyst. Environ..

[bb0040] Grant R.F. (1995). Dynamics of energy, water, carbon and nitrogen in agricultural ecosystems - simulation and experimental validation. Ecol. Model..

[bb0045] Harrod T.R., Hogan D.V. (2008). The Soils of North Wyke and Rowden.

[bb0050] Humphreys M.W., Yadav R.S., Cairns A.J., Turner L.B., Humphreys J., Skot L. (2006). A changing climate for grassland research. New Phytol..

[bb0055] Humphreys M.W., O'Donovan G., Sheehy-Skeffington M. (2014). Comparing synthetic and natural grasslands for agricultural production and ecosystem service. Futur. Eur. Grassl..

[bb0060] Kell D.B. (2011). Breeding crop plants with deep roots: their role in sustainable carbon, nutrient and water sequestration. Ann. Bot..

[bb0065] Knight D., Elliott P.W., Anderson J.M., Scholefield D. (1992). The role of earthworms in managed, permanent pastures in Devon, England. Soil Biol. Biochem..

[bb0070] Lee M.R.F., Jones E.L., Moorby J.M., Humphreys M.O., Theodorou M.K., MacRae J.C., Scollan N.D. (2001). Production responses from lambs grazed on *Lolium perenne* selected for an elevated water-soluble carbohydrate concentration. Anim. Res..

[bb0075] Lee M.R.F., Harris L.J., Moorby J.M., Humphreys M.O., Theodorou M.K., MacRae J.C., Scollan N.D. (2002). Rumen metabolism and nitrogen flow to the small intestine in steers offered *Lolium perenne* containing different levels of water-soluble carbohydrate. Anim. Sci..

[bb0080] Loades K.W., Bengough A.G., Bransby M.F., Hallett P.D. (2010). Planting density influence on fibrous root reinforcement of soils. Ecol. Eng..

[bb0085] Macleod C.K., Humphreys M.W., Whalley W.R., Turner L., Binley A., Watts C.W., Skot L., Joynes A., Hawkins S., King I.P., O'Donovan S., Haygarth P.M. (2013). A novel grass hybrid to reduce flood generation in temperate regions. Sci. Rep..

[bb0090] Marshall A.H., Collins R.P., Humphreys M.W., Scullion J. (2016). A new emphasis on root traits for perennial grass and legume varieties with environmental and ecological benefits. Food Energy Secur..

[bb0095] Merry R.J., Lee M.R.F., Davies D.R., Dewhurst R.J., Moorby J.M., Scollan N.D., Theodorou M.K. (2006). Effects of high-sugar ryegrass silage and mixtures with red clover silage on ruminant digestion. 1. In vitro and in vivo studies of nitrogen utilization. J. Anim. Sci..

[bb0100] Murray P., Crotty F., Eekeren N.V., Wall D.H., Bardgett R.D., Behan-Pelletier V., Herrick J.E., Jones T.H., Karl Ritz J.S., Strong D.R., Puttenfdgd W.H.v.d. (2012). Management of grassland systems, soil, and ecosystem services. Soil Ecology and Ecosystem Services.

[bb0105] Necpálová M., Casey I., Humphreys J. (2013). Effect of ploughing and reseeding of permanent grassland on soil N, N leaching and nitrous oxide emissions from a clay-loam soil. Nutr. Cycl. Agroecosyst..

[bb0110] O'Mara F.P. (2012). The role of grasslands in food security and climate change. Ann. Bot..

[bb0115] Orr R.J., Murray P.J., Eyles C.J., Blackwell M.S., Cardenas L.M., Collins A.L., Dungait J.A., Goulding K.W., Griffith B.A., Gurr S.J., Harris P., Hawkins J.M., Misselbrook T.H., Rawlings C., Shepherd A., Sint H., Takahashi T., Tozer K.N., Whitmore A.P., Wu L., Lee M.R. (2016). The North Wyke Farm Platform: effect of temperate grassland farming systems on soil moisture contents, runoff and associated water quality dynamics. Eur. J. Soil Sci..

[bb0120] Ostle N.J., Levy P.E., Evans C.D., Smith P. (2009). UK land use and soil carbon sequestration. Land Use Policy.

[bb0125] Perego A., Wu L., Gerosa G., Finco A., Chiazzese M., Amaducci S. (2016). Field evaluation combined with modelling analysis to study fertilizer and tillage as factors affecting N_2_O emissions: a case study in the Po valley (Northern Italy). Agric. Ecosyst. Environ..

[bb0130] Peukert S., Griffith B.A., Murray P.J., Macleod C.J., Brazier R.E. (2016). Spatial variation in soil properties and diffuse losses between and within grassland fields with similar short-term management. Eur. J. Soil Sci..

[bb0135] Pierret A., Maeght J.L., Clement C., Montoroi J.P., Hartmann C., Gonkhamdee S. (2016). Understanding deep roots and their functions in ecosystems: an advocacy for more unconventional research. Ann. Bot..

[bb0140] Rumpel C., Creme A., Ngo P., Velasquez G., Mora M., Chabbi A. (2015). The impact of grassland management on biogeochemical cycles involving carbon, nitrogen and phosphorus. J. Soil Sci. Plant Nutr..

[bb0145] Scholefield D., Tyson K.C., Garwood E.A., Armstrong A.C., Hawkins J., Stone A.C. (1993). Nitrate leaching from grazed grassland lysimeters: effects of fertilizer input, field drainage, age of sward and patterns of weather. Eur. J. Soil Sci..

[bb0150] Semmartin M., Di Bella C., de Salamone I.G. (2010). Grazing-induced changes in plant species composition affect plant and soil properties of grassland mesocosms. Plant Soil.

[bb0155] Shepherd M.A., Hatch D.J., Jarvis S.C., Bhogal A. (2001). Nitrate leaching from reseeded pasture. Soil Use Manag..

[bb0160] Shepherd A., Wu L.H., Chadwick D., Bol R. (2011). A review of quantitative tools for assessing the diffuse pollution response to farmer adaptations and mitigation methods under climate change. Adv. Agron..

[bb0165] Smith P., Smith J.U., Powlson D.S., McGill W.B., Arah J.R.M., Chertov O.G., Coleman K., Franko U., Frolking S., Jenkinson D.S., Jensen L.S., Kelly R.H., Klein-Gunnewiek H., Komarov A.S., Li C., Molina J.A.E., Mueller T., Parton W.J., Thornley J.H.M., Whitmore A.P. (1997). A comparison of the performance of nine soil organic matter models using datasets from seven long-term experiments. Geoderma.

[bb0170] Staerfl S.M., Amelchanka S.L., Kalber T., Soliva C.R., Kreuzer M., Zeitz J.O. (2012). Effect of feeding dried high-sugar ryegrass (‘AberMagic’) on methane and urinary nitrogen emissions of primiparous cows. Livest. Sci..

[bb0175] Stokes A., Atger C., Bengough A.G., Fourcaud T., Sidle R.C. (2009). Desirable plant root traits for protecting natural and engineered slopes against landslides. Plant Soil.

[bb0180] Thornley J.H.M., Bergelson J., Parsons A.J. (1995). Complex dynamics in a carbon-nitrogen model of a grass legume pasture. Ann. Bot..

[bb0185] Velthof G.L., Hoving I.E., Dolfing J., Smit A., Kuikman P.J., Oenema O. (2009). Method and timing of grassland renovation affects herbage yield, nitrate leaching, and nitrous oxide emission in intensively managed grasslands. Nutr. Cycl. Agroecosyst..

[bb0190] Vickery J.A., Tallowin J.R., Feber R.E., Asteraki E.J., Atkinson P.W., Fuller R.J., Brown V.K. (2001). The management of lowland neutral grasslands in Britain: effects of agricultural practices on birds and their food resources. J. Appl. Ecol..

[bb0195] Watanabe M.D.B., Ortega E. (2011). Ecosystem services and biogeochemical cycles on a global scale: valuation of water, carbon and nitrogen processes. Environ. Sci. Pol..

[bb0200] Wu L., McGechan M.B., McRoberts N., Baddeley J.A., Watson C.A. (2007). SPACSYS: integration of a 3D root architecture component to carbon, nitrogen and water cycling-model description. Ecol. Model..

[bb0205] Wu L., Rees R.M., Tarsitano D., Zhang X., Jones S.K., Whitmore A.P. (2015). Simulation of nitrous oxide emissions at field scale using the SPACSYS model. Sci. Total Environ..

[bb0210] Wu L., Zhang X., Griffith B.A., Misselbrook T.H. (2016). Sustainable grassland systems: a modelling perspective based on the North Wyke Farm Platform. Eur. J. Soil Sci..

[bb0215] Zhang X.B., Xu M.G., Sun N., Xiong W., Huang S.M., Wu L.H. (2016). Modelling and predicting crop yield, soil carbon and nitrogen stocks under climate change scenarios with fertiliser management in the North China Plain. Geoderma.

