# Supplementary material for: Assessment of soil water, carbon and nitrogen cycling in reseeded grassland on the North Wyke Farm Platform using a process-based model
Source: Sci Total Environ. 2017 Dec 15;603-604:27–37. doi: 10.1016/j.scitotenv.2017.06.012 (PMC5535641; doi:10.1016/j.scitotenv.2017.06.012)
Supplement: Supplementary file 1 — Supplementary tables [file mmc1.docx]

**Appendix. Supplementary information**

Table A1. Soil physical and chemical properties in the top 10cm of soil for individual fields measured in 2012.

| Field | Bulk density (g cm^-3^) | Soil organic matter (g kg^-1^) | Total organic carbon (g kg^-1^) | Total organic nitrogen (g kg^-1^) | pH |
| --- | --- | --- | --- | --- | --- |
| Great Field | 1.03 | 76.68 | 33.12 | 4.08 | 6.40 |
| Longlands East | 0.99 | 96.00 | 37.10 | 4.48 | 6.23 |
| Poor Field | 0.90 | 123.17 | 49.35 | 6.13 | 5.21 |
| Ware Park | 0.86 | 81.00 | 35.18 | 4.56 | 6.37 |
| Pecketsford | 0.95 | 118.42 | 49.49 | 6.33 | 5.38 |
| Little Pecketsford | 0.90 | 126.32 | 52.73 | 6.62 | 5.38 |
| Lower Wheaty | 0.93 | 128.57 | 57.83 | 6.99 | 5.25 |

Table A2: Differences in parameters for the two plant cultivars

| Parameter name | Prior | AberMagic |
| --- | --- | --- |
| Maximum root penetration depth (m) | 0.50 | 0.30 |
| Photosynthate partitioning fraction to root  at vegetative stage (development index: 1.0 - 2.0) |  |  |
| 1.1 | 0.35 | 0.30 |
| 1.2 | 0.35 | 0.25 |
| 1.4 | 0.30 | 0.22 |
| 1.5 | 0.30 | 0.20 |
| 1.7 | 0.25 | 0.20 |
| 2 | 0.05 | 0.15 |
